# Binder-Free Three-Dimensional Porous Graphene Cathodes via Self-Assembly for High-Capacity Lithium–Oxygen Batteries

**DOI:** 10.3390/nano14090754

**Published:** 2024-04-25

**Authors:** Yanna Liu, Wen Meng, Yuying Gao, Menglong Zhao, Ming Li, Liang Xiao

**Affiliations:** 1 School of Chemistry, Chemical Engineering and Life Sciences, Wuhan University of Technology, Wuhan 430070, China; 318495@whut.edu.cn (Y.L.); mengwen@yutong.com (W.M.); gaoyuying@whut.edu.cn (Y.G.); 2 Zhengzhou Yutong Bus Co., Ltd., Zhengzhou 450016, China; 3 School of Materials Science and Engineering, Wuhan University of Technology, Wuhan 430070, China; 335070@whut.edu.cn (M.Z.); 266648@whut.edu.cn (M.L.)

**Keywords:** binder-free cathode, porous graphene, ruthenium, manganese dioxide, lithium–oxygen batteries

## Abstract

The porous architectures of oxygen cathodes are highly desired for high-capacity lithium–oxygen batteries (LOBs) to support cathodic catalysts and provide accommodation for discharge products. However, controllable porosity is still a challenge for laminated cathodes with cathode materials and binders, since polymer binders usually shield the active sites of catalysts and block the pores of cathodes. In addition, polymer binders such as poly(vinylidene fluoride) (PVDF) are not stable under the nucleophilic attack of intermediate product superoxide radicals in the oxygen electrochemical environment. The parasitic reactions and blocking effect of binders deteriorate and then quickly shut down the operation of LOBs. Herein, the present work proposes a binder-free three-dimensional (3D) porous graphene (PG) cathode for LOBs, which is prepared by the self-assembly and the chemical reduction of GO with triblock copolymer soft templates (Pluronic F127). The interconnected mesoporous architecture of resultant 3D PG cathodes achieved an ultrahigh capacity of 10,300 mAh g^−1^ for LOBs. Further, the cathodic catalysts ruthenium (Ru) and manganese dioxide (MnO_2_) were, respectively, loaded onto the inner surface of PG cathodes to lower the polarization and enhance the cycling performance of LOBs. This work provides an effective way to fabricate free-standing 3D porous oxygen cathodes for high-performance LOBs.

## 1. Introduction

The large-scale application of electric and hybrid vehicles has led to an increasing demand for advanced power sources with high energy densities. Therefore, in the past decade, rechargeable lithium–oxygen batteries (LOBs) have prompted intensive studies because of their ultrahigh theoretical energy density of 3500 W h kg^−1^, which is more than ten times the commercially available lithium-ion batteries [1,2,3,4]. A LOB is typically assembled by a lithium–metal anode, a glass fiber separator soaked with organic electrolyte, and a porous oxygen diffusion electrode exposed to gaseous O_2_ during cell operation. At the discharge of LOB, O_2_ is reduced to insoluble Li_2_O_2_ in the cathode with the presence of lithium ions in the electrolyte. The discharge product Li_2_O_2_ accumulates in the porous cathode, blocking the electrolyte and oxygen diffusion paths and causing the cell to shut down suddenly [5,6]. Therefore, rationally designed porous architectures of oxygen cathodes are required to accommodate discharge products and maintain the mass diffusion and discharge/charge process [7]. Besides the issue of insoluble Li_2_O_2_ deposition, the oxygen reduction reaction (ORR) and oxygen evolution reaction (OER) in aprotic electrolytes are recognized to be intrinsically sluggish [8]. In particular, the solid product Li_2_O_2_ needs to ultimately contact the cathode catalyst to be oxidized at the charge of LOB. Thus, rationally designed porous architectures of cathodes are also required to ensure the contact of Li_2_O_2_ and catalysts and the full utilization of catalysts [9,10].

In previous studies, the porous architecture of cathodes for LOBs was commonly fabricated by carbon materials with high specific surface areas and conductivities [11,12,13,14,15]. Typically, a slurry containing a conductive porous carbon powder, a polymer binder, and an optional catalyst is spread onto a piece of conductive carbon paper to prepare the cathodes of LOBs. In this process, the polymer binders partially obstruct the active sites of catalysts and cathode pores, which will deteriorate the catalyst utilization and cathode porosity. The oxygen cathodes prepared by slurry coating usually result in low capacities and high polarizations of LOBs [9,16]. Additionally, polymer binders such as poly(vinylidene fluoride) (PVDF) are not stable under the nucleophilic attack of intermediate product superoxide radicals in the oxygen electrochemical environment [17,18]. The rapid accumulation of parasitic reactions from binders could result in the rapid degradation of cathodes, which in turn leads to performance deterioration in LOBs. Therefore, fabricating three-dimensional (3D) porous oxygen cathodes without binders and with controllable porosity is vital to enhancing LOB’s electrochemical properties.

Due to the specific porous architecture derived from the aligned nanostructures, various metal oxide nanoarrays have been prepared as efficient binder-free porous cathodes to lower the polarization and enhance the cycling performance of LOBs [19,20,21]. However, the limited mass loading of metal oxide nanoarrays on current collectors usually results in low reversible capacities of oxygen cathodes, which thus hinders the full demonstration of the ultrahigh theoretical energy densities of LOBs. Therefore, to achieve high practical capacities of LOBs, it is desirable to use innovative materials with high electrical conductivity and specific surface area for preparing binder-free oxygen cathodes. In the past decade, graphene materials have been intensively studied as innovative functional materials for energy storage applications [22,23]. Graphene materials have high specific surface areas and ultrahigh electronic conductivities due to their two-dimensional (2D) structures of connected sp^2^-hybridized carbon atoms, enabling them promising cathode materials for LOBs [24,25,26,27]. Moreover, it is promising to produce abundant catalytically active sites for oxygen cathodes by modifying the surface functional groups and introducing heteroatoms to graphene materials [27,28]. More importantly, 2D graphene sheets can self-assemble to form a favorable 3D hierarchical porous architecture for the oxygen cathodes of LOBs. These features make graphene an ideal material for fabricating binder-free porous cathodes [29].

Currently, a variety of methods have been reported for the efficient preparation of freestanding binder-free porous graphene electrodes. For example, graphene foams have been prepared as porous cathodes for LOBs by the carbonization of polymer precursors including melamine foam [28], polyacrylonitrile [29], etc. Graphene foams as porous cathodes have been also prepared via the electrochemical leavening of graphite papers [30]. The CVD method has been used to deposit graphene on hard templates, and porous graphene electrodes have been successfully prepared after removing the templates [4,31,32]. Moreover, 3D graphene aerogels as porous cathodes of LOBs have been prepared by hydrothermal routes [33,34]. In addition to the abovementioned physical and chemical methods, self-assembling graphene nanosheets is a simple and controllable way to prepare porous graphene electrodes. Generally, graphene oxides (GOs) and reduced graphene oxides (rGOs), containing oxygenate groups and defects, can self-assemble to form 3D porous architectures by the major driving forces of hydrogen bonds and π–π stacking interactions [35,36]. Due to the restoration of the π-conjugation on the graphene layer via the complete reduction, graphene electrodes with 3D porous architectures obtain high electrical conductivity and flexibility.

During the chemical self-assembly of graphene materials, hard/soft templates are usually used to provide continuously interconnected 3D structures. After the chemical reduction of GO and template removal, if necessary, 3D self-assembly porous graphene (PG) frameworks form an ideal oxygen cathode for LOBs. According to the literature, PG materials can be classified into monolayer PG with in-plane pores, stacked 2D laminar PG with interlayer pores, and 3D interconnected porous frameworks. In the present study, without other specifications, PG cathodes mean 3D porous frameworks of self-assembled graphene materials. For instance, porous graphene paper as the cathode for LOBs has been prepared by vacuum-assisted technique and mixing graphene and poly(4-styrenesulfonic acid) stabilizers [37]. Among alternative template materials, triblock copolymer Pluronic F127 is a versatile and commonly used soft template for the preparation of orderly mesoporous carbon materials and electrodes [38]. Inspired by the preparation of mesoporous carbon, herein, the present work attempted to prepare binder-free 3D PG cathodes for LOBs by the self-assembly and chemical reduction of GO with triblock copolymer Pluronic F127 as the soft template. Experimental results demonstrated that resultant free-standing 3D PG cathodes consisted of interconnected mesoporous architecture, which achieved an ultrahigh capacity of 10,300 mAh g^−1^ for LOBs. Further, the porous architecture of 3D PG cathodes was demonstrated to be suitable for the loading of cathodic catalysts ruthenium (Ru) and manganese dioxide (MnO_2_). This work provides an effective way to fabricate free-standing 3D porous oxygen cathodes for the enhanced performance of LOBs.

## 2. Materials and Methods

### 2.1. Preparation of PG and Catalyst-Decorated PG Cathode

In this study, 3D PG cathodes for LOBs were prepared by the chemical reduction and self-assembly of GO with triblock copolymer soft templates (Pluronic F127). First, 3 mL GO dispersion (1 mg·mL^−1^, Shanxi Institute of Coal Chemistry, Chinese Academy of Sciences) was heated to 95 °C in a 50 mL round-bottomed flask. Next, 15 μL of hydrazine hydrate solution (85% mass fraction) was added, and the mixture was stirred continuously for 1 h. After that, a 10 wt.% Pluronic F127 aqueous solution was added into the flask as the soft template, and stirring was continued for 2 h. Finally, 1.5 mL of concentrated hydrochloric acid was added to the solution, and then the solution was stirred for 2 h. The flask was cooled down to room temperature to obtain a suspension. The suspension was vacuum-filtered onto a piece of carbon paper (Tori) to prepare the 3D PG membranes. The as-prepared electrodes were annealed in a tube furnace under Ar flow at 350 °C for 2 h and 900 °C for 1 h to obtain the PG cathodes.

The prepared PG cathodes were anodically polarized at 0.05 mA for 5 min in an electrolyte containing 0.01 mol·L^−1^ Mn(Ac)_2_ and 0.02 mol·L^−1^ NH_4_Ac, with a platinum plate as the counter electrode and a saturated calomel electrode (SCE) as the reference electrode. The obtained electrode was then annealed in the air at 350 °C for 2 h to prepare MnO_2_-decorated PG cathodes (MnO_2_@PG). In another preparation, the prepared PG cathodes were immersed in a 0.01 mol·L^−1^ RuCl_3_ solution for 12 h and then dried and annealed in Ar/H_2_ at 350 °C for 2 h to produce Ru-decorated PG (Ru@PG) cathodes. As reference materials, graphene dispersion (reduced graphene oxide, rGO) was prepared by hydrazine hydrate reduction without a soft template. The laminated graphene (LG) cathodes were prepared as reference electrodes by casting the slurry of graphene and PVDF binder in a mass ratio of 9:1 on a piece of carbon paper used as both the current collector and the gas diffusion layer. The PG, MnO_2_@PG, Ru@PG, and LG cathodes were vacuum-dried and pouched into round disks with diameters of 15 mm.

### 2.2. Material Characterization

The morphologies of PG, MnO_2_@PG, Ru@PG, and LG cathodes before and after discharge were investigated using optical microscopy and field-emission scanning electron microscopy (SEM, S-4800, Hitachi, Tokyo, Japan). The nitrogen adsorption–desorption data of PG were measured with a Quantachrome Autosorb–1 analyzer at −196 °C. The surface areas were calculated by the Brunauer–Emmett–Teller (BET) method. The pore size distributions were derived from the adsorption branches of the isotherms using the Barrett–Joyner–Halenda (BJH) model. The microstructure of MnO_2_@PG and Ru@PG was investigated by transmission electron microscopy (TEM, JEM-2100F, JEOL Ltd., Tokyo, Japan) with an operating voltage of 200 kV. The chemical compositions of MnO_2_@PG and Ru@PG cathodes were characterized by X-ray photoelectron energy spectroscopy (XPS, VG-Multilab 2000, VG Scientific, Waltham, MA, USA) in a vacuum of 2 × 10^−8^ Pa with an Al Kα excitation source. Raman spectra of PG and LG cathodes were obtained with a Raman spectrophotometer (Invia, Renishaw, New Mills, UK).

### 2.3. Electrochemical Performance of LOBs

LOBs were assembled with an anode of lithium foil (with a diameter of 1.5 cm and a thickness of 1 mm), a glass fiber separator (GF/D, Whatman, Maidstone, UK), and a graphene-based cathode (PG, MnO_2_@PG, Ru@PG, and LG) into R2032 coin cells. Holes (diameter of 1 mm, 21 holes) were punched in the bottom canister of the coin cells for oxygen flow. Briefly, 1 M lithium bis(trifluoromethane) sulfonamide (LiTFSI) in tetraethylene glycol dimethyl ether (TEGDME) was used as the electrolyte. All operations were carried out in a glove box filled with Argon, and both the H_2_O and O_2_ levels were less than 0.1 ppm. The prepared cells were transferred into an oxygen container with a pressure of 1 atm. The galvanostatic discharge/charge and cycling tests of LOBs were conducted at room temperature on a Land CT3008W battery testing system. The discharged and recharged cathodes were disassembled from the coin cells and washed with TEGDME several times. The disassembled cathodes were dried and kept in the glove box before SEM and XPS studies.

## 3. Results and Discussion

**Figure 1:**
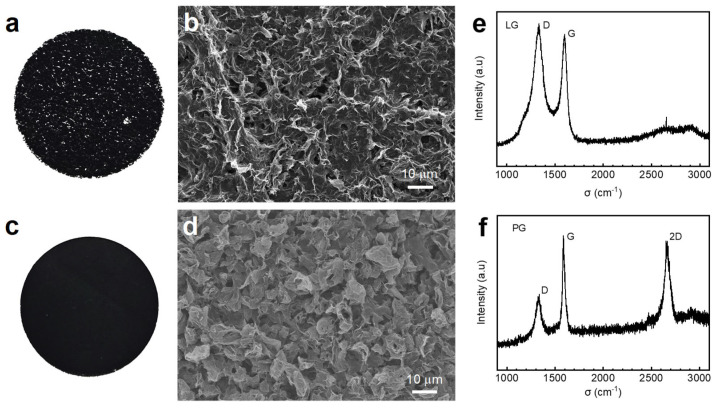
The optical and SEM images of (**a**,**b**) LG and (**c**,**d**) PG cathodes; the Raman spectra of (**e**) LG and (**f**) PG cathodes.

The morphologies and chemical compositions of graphene-based cathodes (LG and PG) prepared by laminating and self-assembly, respectively, were studied by optical microscopy, SEM, and Raman spectroscopy. The laminated graphene cathode was studied as a reference. As shown in Figure 1a, the optical image of LG cathodes prepared using a lamination process with binders shows a rough surface due to the uneven distribution of graphene aggregations and binders. The SEM image in Figure 1b confirms that the binders in LG cathodes enclose the aggregations of graphene sheets, resulting in the blockage of porous structure and uneven pore distribution. By contrast, the optical image of PG cathodes in Figure 1c shows a very smooth surface that is assembled by graphene without binders. The further study of PG cathodes under SEM in Figure 1d suggests that the smooth surface is assembled by wrinkled or curly graphene sheets. As studied in Figure S1 of the Supplementary Materials, the rGO sheets usually form cabbage-like morphologies due to the presence of nanoscale wrinkles and defects in the basal plane of graphene [39,40]. Therefore, with Pluronic F127 as a soft template, the wrinkled graphene self-assembled to a 3D porous architecture [41,42]. The 3D porous PG cathodes provide interconnected channels for mass diffusion and the accommodation of discharge products, making it an appropriate cathode for LOBs. The 3D porous architectures of PG cathodes were also confirmed by nitrogen adsorption–desorption tests. As presented in Figure S2 in the Supplementary Materials, the type IV isotherm and uniform BJH pore size distribution of the PG cathode exhibit the characteristics of ordered mesoporous architectures. The BET surface area of the PG cathode is 47.42 m^2^ g^−1^, and the PG cathode possesses a narrow pore size distribution around 3.5 nm. All the experimental results demonstrate that the self-assembly of graphene sheets forms uniformly a distributed 3D interconnected porous architecture.

To further investigate the chemical compositions of LG and PG cathodes, the Raman spectra of these two cathodes are comparatively studied in Figure 1e,f. The Raman spectrum of the LG cathode in Figure 1e exhibits two remarkable peaks at around 1350 and 1580 cm^−1^, which are assigned to the D and G bands of graphite, respectively [22,43]. There are no obvious and sharp 2D peaks of single-layer graphene at 2690 cm^−1^, suggesting the multilayer stacking of the graphene layer in LG and the loss of active surfaces. In Figure 1f, the main Raman peaks of single-layer graphene are observed for the PG cathodes. Those are the G band at 1580 cm^−1^, a primary in-plane vibrational mode, and the 2D band at 2690 cm^−1^, a second-order overtone of a different in-plane vibration. Furthermore, the low ratio of peak intensities I_D_/I_G_ indicates an increasing defect density [22,43]. The prepared PG cathodes therefore have structural defects to offer reactive sites for the oxygen reduction reaction (ORR) and the decomposition of Li_2_O_2_ in the LOBs.

**Figure 2:**
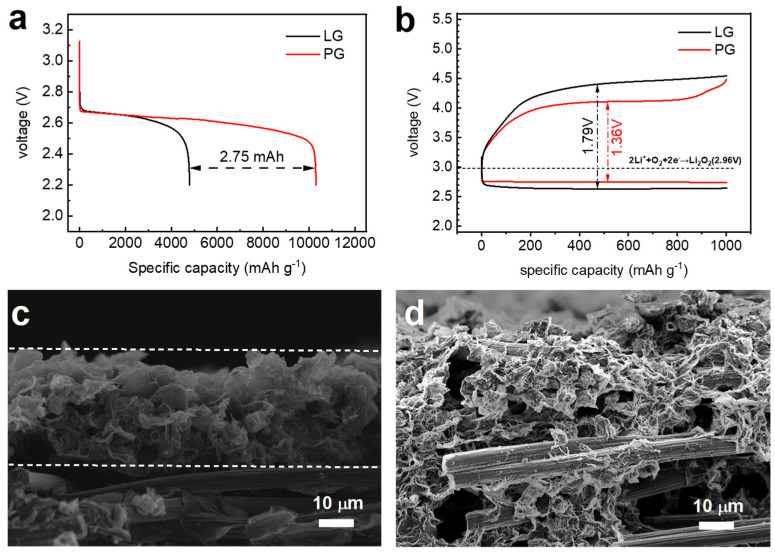
(**a**) The discharge profiles of PG and LG cathodes at a current density of 200 mA g^−1^; (**b**) the voltage profiles of PG and LG cathodes at 200 mA g^−1^ with a curtailed capacity of 1000 mAh g^−1^; the cross-section SEM images of (**c**) PG and (**d**) LG.

The electrochemical performance of LOBs using LG and PG cathodes was studied by constant current discharge and charge. Figure 2a compares the discharge capacities of LG and PG cathodes at the first cycle with a cut-off voltage of 2.2 V at the current density of 200 mA g^−1^. Based on the 0.5 mg cm^−2^ mass loading of graphene materials, LG and PG cathodes demonstrate specific capacities of 4800 mA h g^−1^ and 10,300 mA h g^−1^, respectively. In other words, the capacity difference between LG and PG cathode was calculated to be 2.75 mAh cm^−2^. The 3D porous architecture of the PG cathode provides abundant mass diffusion paths, reactive sites for the ORR, and accommodation for discharge products, resulting in a discharge capacity that is more than two times the capacity of the LG cathode. Compared with the binder-free freestanding carbonaceous oxygen cathodes reported in the literature, the PG cathode in the present work shows almost the highest discharge capacity of 10,300 mA h g^−1^ or 5.15 mA h cm^−2^, as shown in Table S1 in the Supplementary Materials. The electrode process kinetics of LG and PG cathodes including reversibility and electrode polarization were studied by constant current discharge and charge with curtailed capacities. Since the fully discharged graphene cathodes cannot be charged back, Figure 2b compares the voltage profiles of LG and PG cathodes with a curtailed specific capacity of 1000 mAh g^−1^. Benefiting from the 3D porous architecture, the PG cathode has an overall charge–discharge overpotential of 1.36 V, which is significantly lower than that of the LG cathode (1.79 V). The 3D porous architecture of the PG cathode was further studied with cross-sectional SEM images. As shown in Figure 2c, PG shows a self-assembled layer of porous graphene with a thickness of about 40 μm (between the dash lines). By contrast, the cross-section of LG in Figure 2d shows the aggregation of graphene and binder infiltrating into the carbon paper, which deteriorates the full utilization of the graphene surface.

**Figure 3:**
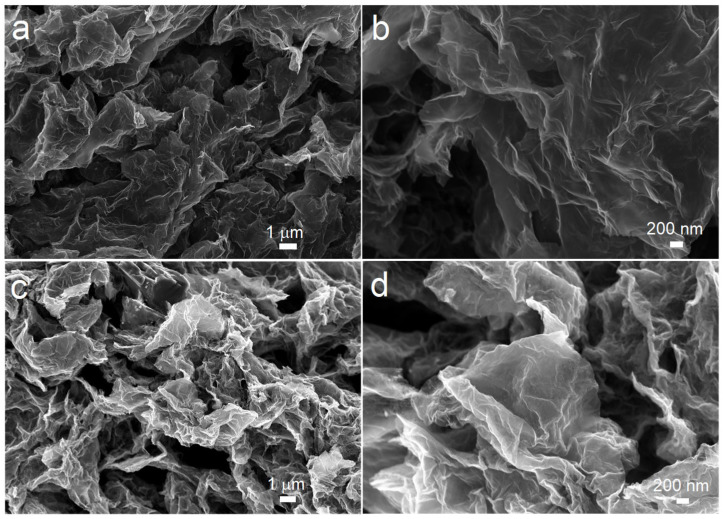
The SEM images of (**a**,**b**) MnO_2_@PG and (**c**,**d**) Ru@PG.

**Figure 4:**
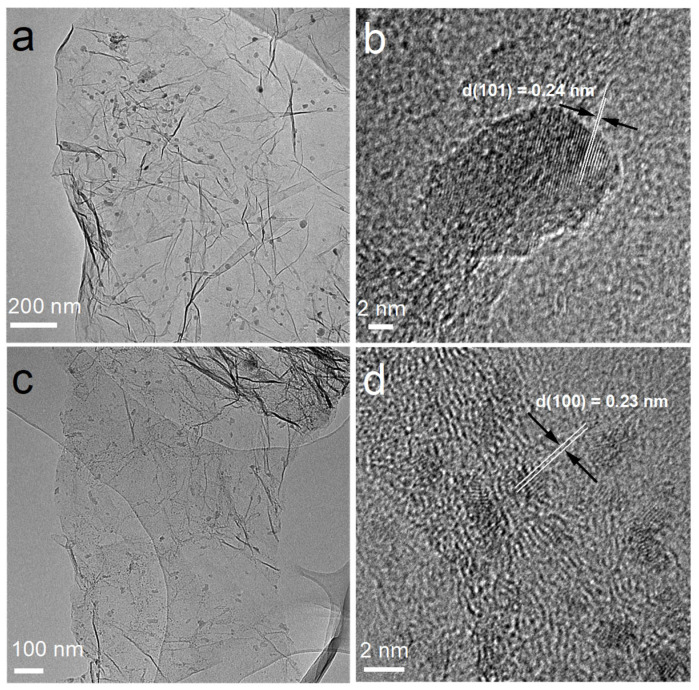
The TEM and HR-TEM images of (**a**,**b**) MnO_2_@PG and (**c**,**d**) Ru@PG.

**Figure 5:**
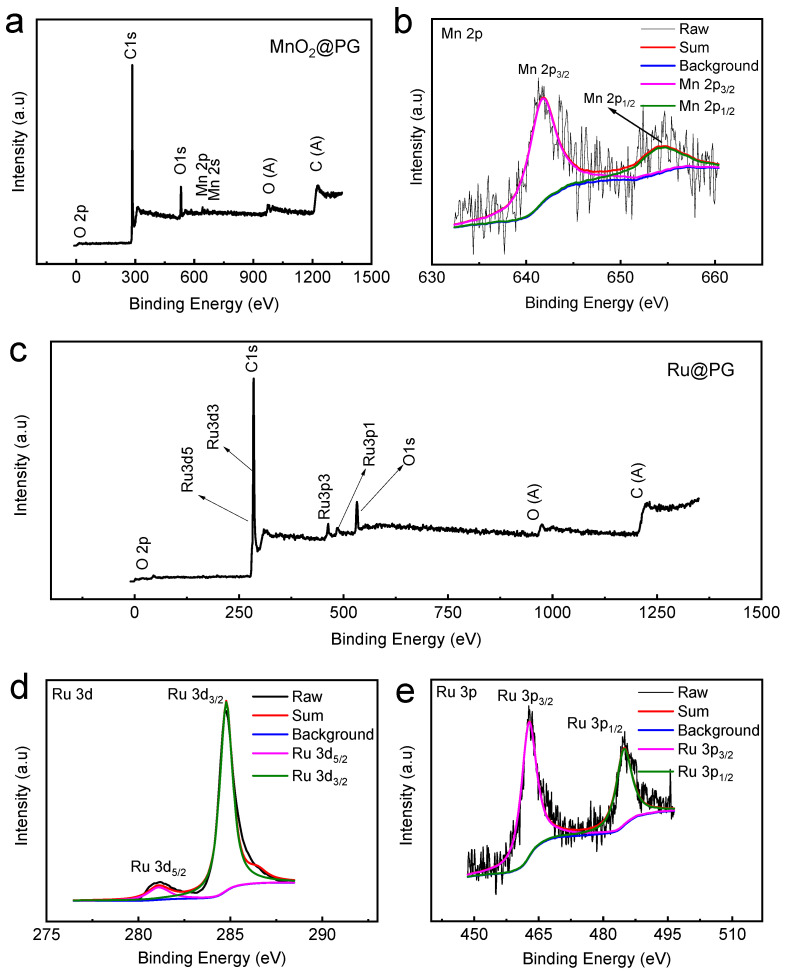
The XPS spectra of MnO_2_@PG and Ru@PG: (**a**) survey and (**b**) Mn 2p spectra of MnO_2_@PG; (**c**) survey, (**d**) Ru 3d, and (**e**) Ru 2p spectra of Ru@PG.

Although the 3D porous architecture of the PG cathode achieves ultrahigh capacities, the catalytically inert nature of graphene gives rise to high charging overpotentials for Li_2_O_2_ oxidation. Even with a curtailed capacity, PG cathodes stably cycle only for tens cycles due to the high polarization (Figure S3a). This result is consistent with previous reports in the literature. The previously reported binder-free freestanding carbonaceous oxygen cathodes for LOBs usually stably function for only tens cycles [4,28,29,30,31,33,34,37,44]. Therefore, to reduce the overpotential of the charging process and achieve better cycle performance, efficient catalysts should be introduced to modify the carbonaceous cathodes. The present study then investigated the ability of PG cathodes to support MnO_2_ and Ru catalysts via their 3D mesoporous architectures. As shown in Figure 3, after the incorporation of catalysts, MnO_2_@PG and Ru@PG maintain the porous architecture of PG cathodes. The introduction of catalysts onto the cathode surface does not change the morphologies of PG-based cathodes. The more detailed morphologies and crystalline structures of MnO_2_@PG and Ru@PG were investigated by TEM and HRTEM, and the results are shown in Figure 4. The TEM image in Figure 4a shows that MnO_2_ nanosheets are evenly distributed on the graphene sheet layer. Further, the HRTEM image of MnO_2_@PG in Figure 4b shows that the interplanar spacing between lattice planes is 0.24 nm, which corresponds to the (101) planes of the β-MnO_2_ structure [45]. Figure 4c shows that small particles of Ru with a size of 5 nm are evenly distributed on the graphene sheet layer. In the HR-TEM image of Ru particles in Figure 4d, the distinct lattice fringes (ascribed to the (100) plane) with a spacing of 0.23 nm verify the successful loading of Ru on Ru@PG further [46]. The chemical compositions of MnO_2_@PG and Ru@PG were further investigated by XPS. The survey spectrum of MnO_2_@PG in Figure 5a shows the presence of Mn, C, and O without other purities. In Figure 5b, the Mn 2p_3/2_ and Mn 2p_1/2_ peaks at 641.8 eV and 653.4 eV are ascribed to Mn(IV) in MnO_2_ [47]. The survey spectrum in Figure 5c shows the presence of C, Ru, and O. The fine Ru 3D spectrum given in Figure 5d exhibits the core-level spectra at 285 eV and 281 eV, corresponding to Ru 3d_3/2_ and Ru 3d_5/2_. Moreover, the binding energy components at 462.7 eV and 484.5 eV in Figure 5e are attributed to Ru 3p_3/2_ and Ru 3p_1/2_, respectively [48]. All the above-mentioned results demonstrate that the porous architecture of PG cathodes is suitable to support dispersed MnO_2_ or Ru catalysts.

**Figure 6:**
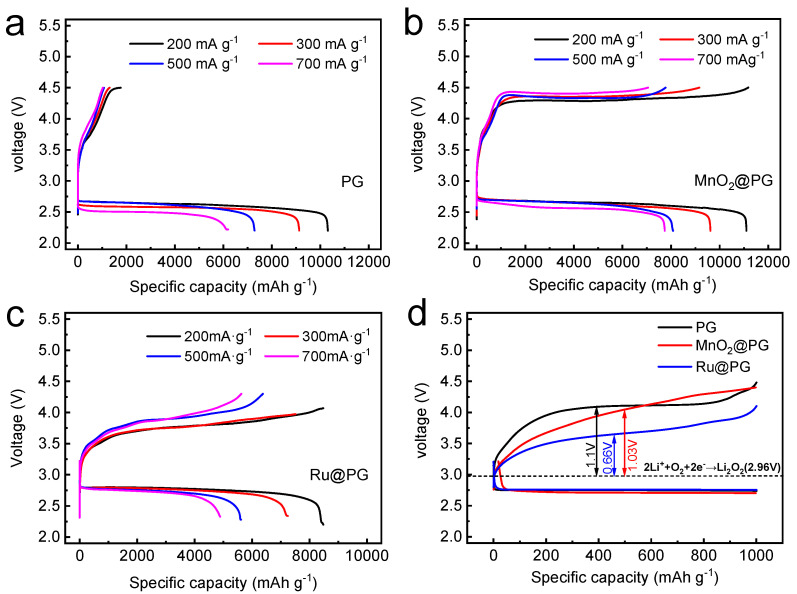
The voltage profiles of LOBs with (**a**) PG, (**b**) MnO_2_@PG, and (**c**) Ru@PG cathodes at different current densities; (**d**) the voltage profiles of PG, MnO_2_@PG, and Ru@PG at 200 mA g^−1^ with a curtailed specific capacity of 1000 mAh g^−1^.

After the loading of MnO_2_ or Ru catalysts, the electrochemical performances of MnO_2_@PG and Ru@PG were studied by the galvanostatic charge and discharge of LOBs from 2.2 to 4.5 V at current densities ranging from 200 mA g^−1^ to 700 mA g^−1^. The voltage profiles of PG, MnO_2_@PG, and Ru@PG are presented in Figure 6a–c, respectively. In Figure 6a, as discussed in Figure 2a, the PG cathode shows high discharge capacities and excellent rate capability due to its 3D porous architecture. The PG cathode delivers a high capacity of 6000 mAh g^−1^ at a high current density of 700 mA g^−1^. However, the PG cathode cannot be charged back with a cut-off voltage of 4.5 V due to its high charge polarization derived from the sluggish OER kinetics. Thus, catalysts including MnO_2_, Ru, etc., should be utilized to suppress anodic polarizations. With the loading of MnO_2_, as shown in Figure 6b, MnO_2_@PG has a slightly larger capacity than PG, which might be ascribed to the capacity contribution of nanostructured MnO_2_. Moreover, the MnO_2_ catalyst enables the charge/discharge reversibility of MnO_2_@PG from 2.2 to 4.5 V at current densities of 200, 300, 500, and 700 mA g^−1^, although the charge polarizations are still high. At a high current density of 700 mA g^−1^, MnO_2_@PG still delivers a high reversible capacity of 7735 mAh g^−1^. Further studies shown in Figure 6c suggest that the loading of Ru extremely decreases the charge and discharge polarization of Ru@PG. Therefore, Ru is an appropriate catalyst for the PG cathode. It should also be noticed that the capacities of Ru@PG are slightly lower than PG and MnO_2_@PG. The loading of Ru onto PG might cause the surface area loss of PG to some extent. The OER catalytical activities of MnO_2_ and Ru are further studied by galvanostatic discharge/charge with a curtailed capacity of 1000 mAh g^−1^. As compared in Figure 6d, PG shows the highest anodic polarization of 1.1 V, and MnO_2_@PG and Ru@PG show lower anodic polarizations of 1.03 V and 0.66 V, respectively. The OER catalytical activity of Ru@PG is higher than MnO_2_@PG. Therefore, Ru@PG shows the best cycling performance among all three PG-based cathodes (see the voltage profiles during cycling in Figure S2 in the Supplementary Materials).

**Figure 7:**
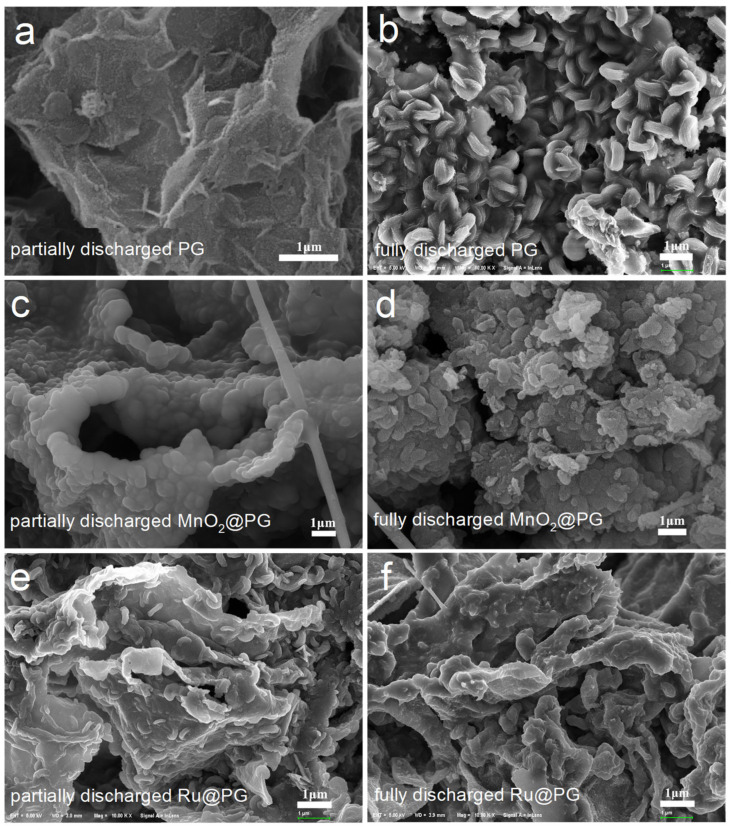
The SEM images of discharged (**a**,**b**) PG, (**c**,**d**) MnO_2_@PG, and (**e**,**f**) Ru@PG cathodes (partially discharged to 1000 mAh g^−1^ and fully discharged to 2.2 V).

**Figure 8:**
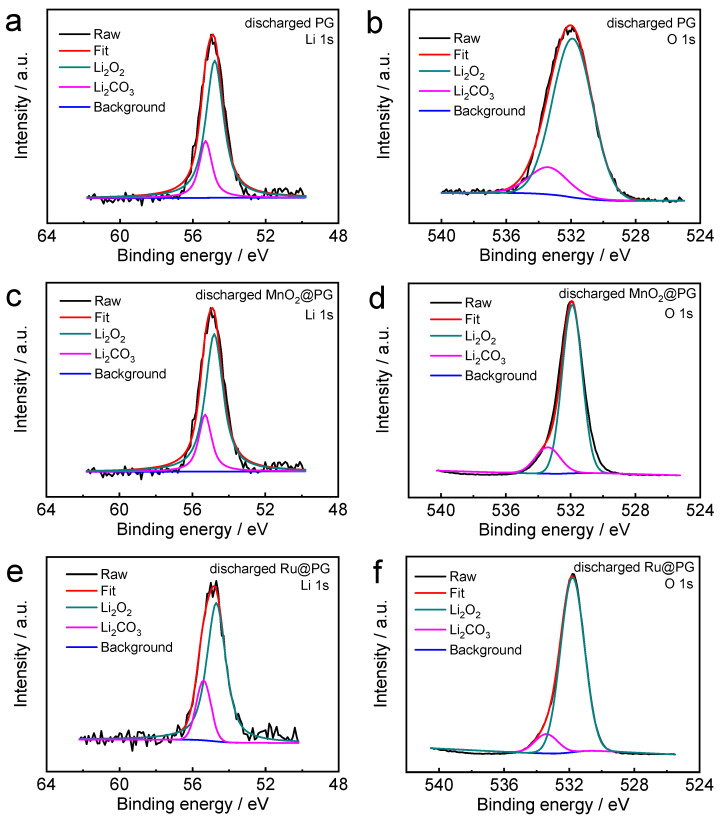
Li 1s and O 1s XPS spectra of discharged cathodes: (**a**,**b**) GMS, (**c**,**d**) MnO_2_@PG, and (**e**,**f**) Ru@PG.

To further illustrate the catalytic activities and discharge mechanisms of MnO_2_@PG and Ru@PG cathodes in LOBs, the discharged MnO_2_@PG and Ru@PG were studied by SEM (Figure 7) and XPS (Figure 8). Figure 7 compares the cathodes discharged to 1000 mAh g^−1^ (marked as partially discharged) and 2.2 V (marked as fully discharged). At the initial stage of PG discharge (shown in Figure 7a), the small particle discharge products evenly deposit on the graphene surface, indicating rich catalytically active sites for ORR on the porous graphene. When discharged to 2.2 V (shown in Figure 7b), the discharge products gradually form toroid-like particles aggregated on the PG cathode to provide the ultrahigh discharge capacity. By contrast, discharged MnO_2_@PG (Figure 7c) shows aggregated particles even in the initial stage of discharge, which could be induced by the nanostructured MnO_2_. The induced growth of discharge products on nanostructured MnO_2_ ensures the ultimate contact between MnO_2_ and discharge product and thus lowers the OER polarization in the following charging process: When discharge to 2.2 V (Figure 7d), MnO_2_@PG shows aggregated particles of discharge products to provide the ultrahigh discharge capacity. The MnO_2_ catalyst actually alters the morphology of discharge products. With the highly active catalyst Ru, discharged Ru@PG shows different morphologies compared to discharged PG and MnO_2_@PG. At the initial stage of discharge, Ru@PG (Figure 7e) already shows small toroid-like discharge particles. Each toroid-like particle could grow around a catalytically active site. When discharged to 2.2 V (Figure 7f), Ru@PG shows the surface conformal growth of discharge products, and no accumulation of product particles is observed. This is why the capacity of Ru@PG is slightly lower than those of PG and MnO_2_@PG. The chemical compositions of discharge PG, MnO_2_@PG, and Ru@PG were studied by XPS. Figure 8 compares the fine XPS spectra of fully discharge cathodes at 200 mA g^−1^ to 2.2 V. Both Li_2_O_2_ and Li_2_CO_3_ can be identified in the Li 1s and O 1s spectra of discharged PG, MnO_2_@PG, and Ru@PG cathodes, and the main XPS peak of discharged cathodes are ascribed to the main product Li_2_O_2_. The presence of Li_2_CO_3_ is ascribed to the degradation of the carbon-based cathodes under oxygen reduction circumstances, as well as to the short exposure to the ambient air before loading into the XPS instrument.

## 4. Conclusions

In the present work, we successfully prepared a binder-free three-dimensional (3D) porous graphene (PG) cathode for LOBs by the self-assembly and chemical reduction of graphene oxide (GO) with triblock copolymer soft templates (Pluronic F127). The interconnected mesoporous architecture of resultant 3D PG cathodes achieved an ultrahigh capacity of 10,300 mAh g^−1^ for LOBs. Further, the porous architecture of PG cathodes was found to be suitable for the uniform loading of cathodic catalysts ruthenium (Ru) and manganese dioxide (MnO_2_). MnO_2_@PG and Ru@PG showed lower levels of anodic polarization, with 1.03 V and 0.66 V, respectively, compared to the anodic polarization of 1.1 V for PG without catalysts. This work provides an effective way to fabricate free-standing 3D porous oxygen cathodes for the enhanced performance of LOBs.

## Data Availability

The datasets generated during and/or analyzed during the current study are available from the corresponding author upon reasonable request.

## Supplementary Materials

The following supporting information can be downloaded at: https://www.mdpi.com/article/10.3390/nano14090754/s1, Figure S1: The SEM images of rGO; Figure S2: (a) The nitrogen sorption isotherm and (b) BJH pore size distribution of PG cathode; Figure S3: The voltages profiles of (a) PG, (b) MnO_2_@PG, and (c) Ru@PG at 200 mA g^−1^ with a curtailed capacity of 1000 mAh g^−1^; Table S1: Discharge capacity comparison of binder-free freestanding carbonaceous oxygen cathodes for LOBs.
