# Supplementary material for: Binder-Free Three-Dimensional Porous Graphene Cathodes via Self-Assembly for High-Capacity Lithium–Oxygen Batteries
Source: Nanomaterials (Basel). 2024 Apr 25;14(9):754. doi: 10.3390/nano14090754 (PMC11085291; doi:10.3390/nano14090754)
Supplement: Supplementary file 1 [file nanomaterials-14-00754-s001.zip › nanomaterials-2954479-supplementary.pdf]

Supplementary Materials

Article

Binder-Free Three-Dimensional Porous Graphene Cathodes via Self-Assembly for High-Capacity Lithium−Oxygen Batteries

Yanna Liu [1,†], Wen Meng [1,3,†], Yuying Gao [1], Menglong Zhao [2], Ming Li [2] and Liang Xiao [1,2,*]

[1] School of Chemistry, Chemical Engineering and Life Sciences, Wuhan University of Technology,
Wuhan 430070, China; gaoyuying@whut.edu.cn (Y.G.)
[2] School of Materials Science and Engineering, Wuhan University of Technology, Wuhan 430070, China; 266648@whut.edu.cn (M.L.)
[3] Zhengzhou Yutong Bus Co., LTD., Zhengzhou, 450016, China
* Correspondence: xiaoliang@whut.edu.cn (L.X.)
† These authors contributed equally to this work.

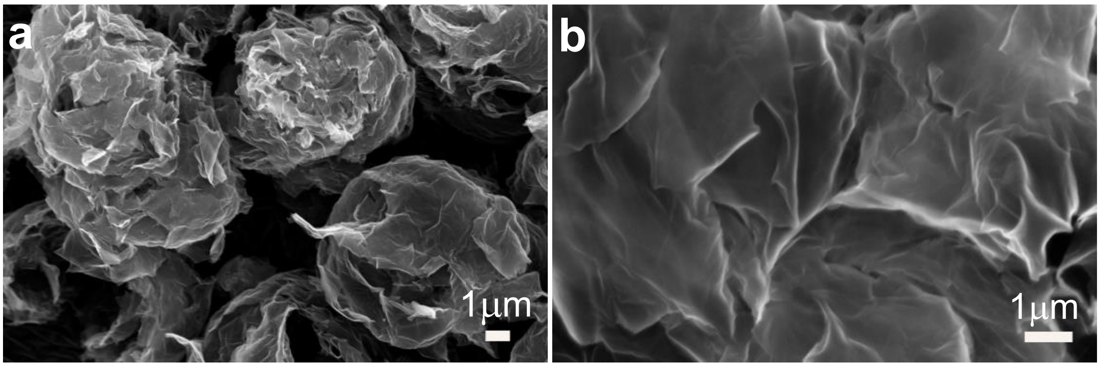

Figure S1. The SEM images of rGO.

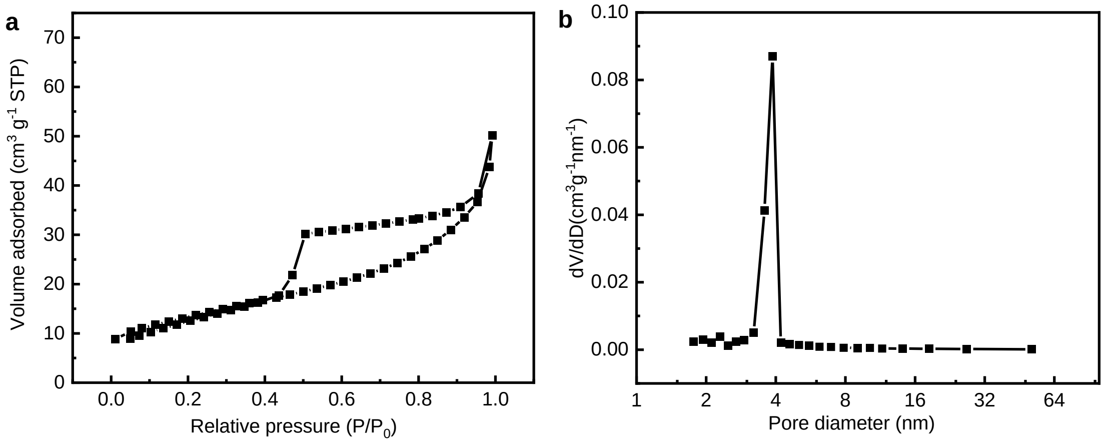

Figure S2. (a) The nitrogen sorption isotherm and (b) BJH pore size distribution of

PG cathode.

Table S1. Discharge capacity comparison of binder-free freestanding carbonaceous oxygen cathodes for LOBs.

Materials	Synthesis	Discharge capacity (discharge rate)	Year [Reference]
3D porous graphene	Self-assembling with F127 templates	10300 mAh g^{-1} (200 mA g^{-1}, 0.1 mA cm^{-2})	The present work
Graphene foam	electrochemical leavening of the graphite papers	340 mAh g^{-1} (100 mA g^{-1} at the 20th cycle)	2013 [1]
3D graphene aerogel	Hydrothermal route	236 mAh g^{-1} (50 mA g^{-1})	2014 [2]
Graphene paper	vacuum-assisted technique by mixing graphene with poly(4-styrenesulfonic acid) stabilizers	6910 mAh g^{-1} (200 mA g^{-1})	2015 [3]
Graphene aerogel	Hydrothermal route	10000 mAh g^{-1} (0.1 mA cm^{-2})	2016 [4]
Graphene foam	Carbonized melamine foam	3200 mAh g^{-1} (100 mA g^{-1})	2016 [5]
CNTs-grafted graphene foam	Chemical vapor deposition	10300 mAh g^{-1} (0.1 mA cm^{-2})	2018 [6]
Freestanding carbon nanotube films	Conversion from superaligned CNT arrays	4050 mAh g^{-1} (0.1 mA cm^{-2})	2018 [7]
Graphite foam	polyacrylonitrile graphitization	1.2 mAh cm^{-2} (0.1 mA cm^{-2})	2021 [8]
Hierarchically porous graphene	Al_2O_3 hard template and CVD method	6300 mAh g^{-1} 0.4 mA cm^{-2}	2024 [9]

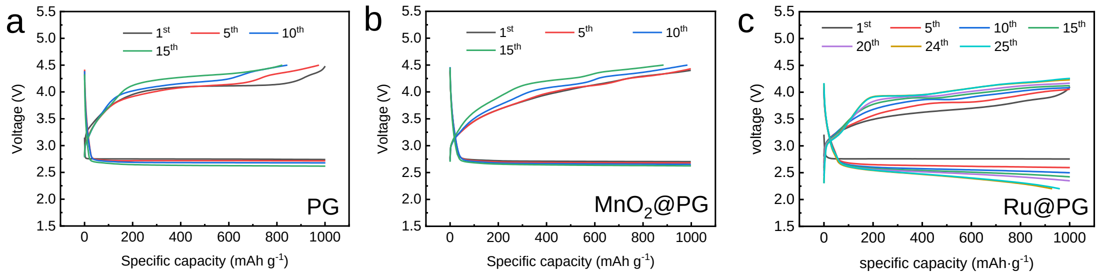

Figure S3. The voltages profiles of (a) PG, (b) MnO_2@PG, and (c) Ru@PG at 200 mA g^{-1} with a curtailed capacity of 1000 mAh g^{-1}.

References:

1. Zhang, W.; Zhu, J.; Ang, H.; Zeng, Y.; Xiao, N.; Gao, Y.; Liu, W.; Hng, H.H.; Yan, Q. Binder-free graphene foams for O2 electrodes of Li–O2 batteries. *Nanoscale* **2013**, *5*, 9651-9658, doi:10.1039/C3NR03321J.
2. Zhu, X.; Zhang, P.; Xu, S.; Yan, X.; Xue, Q. Free-Standing Three-Dimensional Graphene/Manganese Oxide Hybrids As Binder-Free Electrode Materials for Energy Storage Applications. *ACS Appl. Mater. Interfaces* **2014**, *6*, 11665-11674, doi:10.1021/am5024258.
3. Kim, D.Y.; Kim, M.; Kim, D.W.; Suk, J.; Park, O.O.; Kang, Y. Flexible binder-free graphene paper cathodes for high-performance Li-O2 batteries. *Carbon* **2015**, *93*, 625-635, doi:https://doi.org/10.1016/j.carbon.2015.05.097.
4. Jiang, J.; He, P.; Tong, S.; Zheng, M.; Lin, Z.; Zhang, X.; Shi, Y.; Zhou, H. Ruthenium functionalized graphene aerogels with hierarchical and three-dimensional porosity as a free-standing cathode for rechargeable lithium-oxygen batteries. *NPG Asia Materials* **2016**, *8*, e239-e239, doi:10.1038/am.2015.141.
5. Zhang, P.; Wang, R.; He, M.; Lang, J.; Xu, S.; Yan, X. 3D Hierarchical Co/CoO-Graphene-Carbonized Melamine Foam as a Superior Cathode toward Long-Life Lithium Oxygen Batteries. *Adv. Funct. Mater.* **2016**, *26*, 1354-1364, doi:https://doi.org/10.1002/adfm.201503907.
6. Wan, W.; Zhu, X.; He, X.; Wang, Y.; Yan, Y.; Wu, Y.; Lü, Z. Nanoarchitectured CNTs-Grafted Graphene Foam with Hierarchical Pores as a Binder-Free Cathode for Lithium-Oxygen Batteries. *J. Electrochem. Soc.* **2018**, *165*, A1741, doi:10.1149/2.1041809jes.
7. Yu, W.; Wang, H.; Qin, L.; Hu, J.; Liu, L.; Li, B.; Zhai, D.; Kang, F. Controllable Electrochemical Fabrication of KO2-Decorated Binder-Free Cathodes for Rechargeable Lithium–Oxygen Batteries. *ACS Appl. Mater. Interfaces* **2018**, *10*, 17156-17166, doi:10.1021/acsami.8b02359.
8. Yang, L.; Chen, J.; Xu, S.; Jing, N.; Hao, H.; Wang, Z.; Wang, M.; Wang, G.; Wang, G. Binder-Free Flexible Three-Dimensional Porous Electrodes by Combining Microstructures and Catalysis to Enhance the Performance of Lithium-Oxygen Batteries. *Ind. Eng. Chem. Res.* **2021**, *60*, 14113-14123, doi:10.1021/acs.iecr.1c02765.
9. Yu, W.; Shen, Z.; Yoshii, T.; Iwamura, S.; Ono, M.; Matsuda, S.; Aoki, M.; Kondo, T.; Mukai, S.R.; Nakanishi, S.; Nishihara, H. Hierarchically Porous and Minimally Stacked Graphene Cathodes for High-Performance Lithium–Oxygen Batteries. *Adv. Energy Mater.* **2024**, *14*, 2303055, doi:https://doi.org/10.1002/aenm.202303055.
